# Primary leptomeningeal histiocytic lymphoproliferative disorder associated with SARS-CoV-2 brain infection in k18-hACE2 mouse: a case report

**DOI:** 10.1186/s42826-025-00256-4

**Published:** 2025-09-22

**Authors:** Néstor Porras, Lidia Sánchez-Morales, Marta Pérez-Sancho, Lucas Domínguez, Antonio Rodríguez-Bertos

**Affiliations:** 1 VISAVET Health Surveillance Centre, Complutense University of Madrid, Madrid, 28040 Spain; 2 Department of Animal Health, Faculty of Veterinary Medicine, Complutense University of Madrid, Madrid, 28040 Spain; 3 Department of Internal Medicine and Animal Surgery, Faculty of Veterinary Medicine, Complutense University of Madrid, Madrid, 28040 Spain

**Keywords:** Histiocytic neoplasm, K18-hACE2, Immunohistochemistry, Neuroinvasion, SARS-CoV-2

## Abstract

**Background:**

Histiocytic proliferative disorders in the central nervous system are rare, and their potential association with viral infections remains largely unexplored. This case is relevant because it suggests a potential interaction between SARS-CoV-2 neuroinvasion and tumor development, providing insights into how viral infections might influence oncogenesis.

**Case presentation:**

A 4.5-month-old male k18-hACE-2 mouse, part of an experimental study of SARS-CoV-2, displayed a small mass in leptomeningeal area composed by neoplastic round cells. This process is associated with typical acute inflammatory and neurodegenerative lesions according to viral neuroinvasion. Histopathology revealed a well-demarcated tumor composed of lymphoblasts and intermixed with abundant histiocytic-like cells. Immunohistochemistry showed high expression of Iba-1 in histiocytes but negative PAX5, CD3 and IRF-4 labeling. Due to the critical role of PAX-5 in maintaining B-cell function, its reduction or inactivation may favor this loss of identity and differentiation to macrophages, which supports the possibility of a lymphoma undergoing transdifferentiation into a histiocytic/dendritic cells neoplasm. Additionally, SARS-CoV-2 was detected within the tumor histiocytes and adjacent neurons, raising questions about potential interactions between viral infection and tumor development.

**Conclusions:**

While the underlying mechanisms remain uncertain, this finding highlights the need for further investigation into the interplay between SARS-CoV-2 infection and oncogenesis. This case represents the first report of a primary brain histiocytic lymphoproliferative disorder associated with SARS-CoV-2 in k18-hACE2 mouse.

## Background

Severe acute respiratory syndrome coronavirus 2 (SARS-CoV-2) is well known as the causative agent of coronavirus disease in 2019 (COVID-19). SARS-CoV-2 caused numerous neurological complications, including headache and anosmia as the most frequent symptoms. However, more serious complications such as encephalitis, acute disseminated encephalomyelitis, Guillain-Barré syndrome, seizures, delirium, dementia-like syndrome and psychiatric disorders may be observed suggesting neuroinvasion ability [1]. The importance of the brain injure in this disease lies in the fact that 36.4% of patients who died from COVID-19 presented neurological signs and lesions [2]. Neuroinvasion could be related to the presence of high levels of ACE2 receptors in the brain since in humans, it is expressed in the epithelium of kidney, gut, lung, blood vessels and specially, wide present throughout the central nervous system (CNS) (neurons, microglia, astrocytes and oligodendrocytes) [3]. The mice strain used in SARS-CoV-2 studies is the k18-hACE2 mice strain, which expresses the ACE2 gene under the control of the cytokeratin 18 promoter. This strain demonstrated neuroinvasion and encephalitis following SARS-CoV-2 infection, associated with high mortality [4].

Recent studies have indicated that SARS-CoV-2 infection may influence specific molecular and cellular mechanisms involved in cancer initiation and progression, although this relationship remains under active research [5]. Additionally, several studies have reported high severity of SARS-CoV-2 infection in individuals with pre-existing conditions, particularly hematological malignancies [6]. This raises questions about whether SARS-CoV-2 infection could induce or accelerate oncogenic processes.

Primary central nervous system lymphoma (PCNSL) is a highly aggressive non-Hodgkin lymphoma located in CNS, being the 90% of these diffuse large B-cell lymphomas (DLBCL) in humans [7]. In fact, the risk of developing lymphoma is apparently increased in patients with immune disorders such as human immunodeficiency virus (HIV) or disease produced by Epstein-Barr virus (EBV) [8]. Furthermore, the development of tumors, such as lymphomas or leukemias in research animal models can also be associated with retroviruses [9]. Notably, the presence of B-cell non-Hodgkin lymphomas has been reported in transgenic (Tg) mice previously infected with HIV [10].

In this work, we present an unusual finding of a primary tumor resembling a lymphoma transdifferentiating into histiocytic/dendritic neoplasm in the leptomeninges of a k18-hACE2 brain mouse experimentally infected with SARS-CoV-2. Furthermore, we describe for the first time the presence of SARS-CoV-2 within tumor histiocytes by immunohistochemistry, suggesting a possible interaction between viral infection and oncogenesis.

## Case presentation

A 19-week-old male k18-hACE2 mouse was part of a SARS-CoV-2 experimental study [11]. The animal arrived at VISAVET and was challenged with SARS-CoV-2 intranasally (25 µL of 1 × 10^4^ TCID_50_) in BSL3 facilities. Afterwards, animals were weighed daily and monitored for clinical signs (loss of weight, hair appearance, level of activity, eye closure, respiratory and neurological signs) [11]. On the 7th day of post-infection (dpi) the animal was euthanized due to inactivity, closed eyes, and 10% weight loss. Following sacrifice, necropsy and gross examination were conducted, and tissues were collected for the assessment of viral loads and histopathology.

The detection and quantification of SARS-CoV-2 loads from tissues was performed using the CoVID19 dtec RT- qPCR Test (Genetic PCR Solutions™, Alicante, Spain) in which SARS-CoV-2 can be detected at least up to 10 copies with a 100% confidence. Viral loads obtained were 1.65 × 10^5^ copies/µl in brain, 1.20 × 10^2^ copies/µl in lungs and 2.60 × 10^2^ copies/µl in trachea and nasal turbinates. In addition, the same test was carried out to quantify the infection inoculum with a result of 1.94 × 10^6^ copies/µL.

Tissues were fixed in 10% neutral formalin (Panreac AppliChem ITW Reagents, Barcelona, Spain) for 48 h. The samples were automatically processed, embedded in paraffin, stained with hematoxylin-eosin (H&E) and immunohistochemical techniques according to the lab routine rules [11]. Histopathological and immunohistochemical evaluation was performed on consecutive sections of the entire brain. Additionally, histology was performed in all the animal tissues. For immunohistochemistry (IHC), the slides were incubated overnight at 4 °C with the primary antibodies detailed in Table 1. A spleen from a healthy mouse was used as a positive control for PAX5 +, CD3 +, Iba-1 + cells. A spleen from a mouse inoculated with myeloma cells was used as a positive control for IRF-4 + cells. For negative controls, the primary antibody was omitted and substituted by tris-buffered saline. After night, secondary antibody was added (ImmPRESS^®^ VR Horse AntiMouse IGG Polymer Kit, Peroxidase; Vector Laboratories, Newark, California, United States) and incubated for 1 h. For the revealing process peroxidase was used (ImmPACT^®^ NovaRED^®^Substrate Kit Peroxidase; Vector Laboratories, Newark, California, USA). Finally, samples were mounted (CTM6 Coverslipper, Thermo Fisher Scientific) and evaluated for histopathological alterations under a Leica DM2000 microscope (Leica Microsystems, Wetzlar, 162 Germany).

**Table 1:** List and details of antibodies used in the immunohistochemical study

Antibody	Type	Host	Dilution	Company
Anti-SARS-CoV-2	Monoclonal	Mouse	1:100	Thermo Fisher Scientific
Anti-Iba-1	Polyclonal	Rabbit	1:100	Thermo Fisher Scientific
Anti-CD3	Polyclonal	Rabbit	1:100	DAKO
Anti-PAX5	Monoclonal	Rabbit	1:100	Abcam
Anti-IRF-4	Monoclonal	Rabbit	1:100	Abcam

**Fig. 1:**
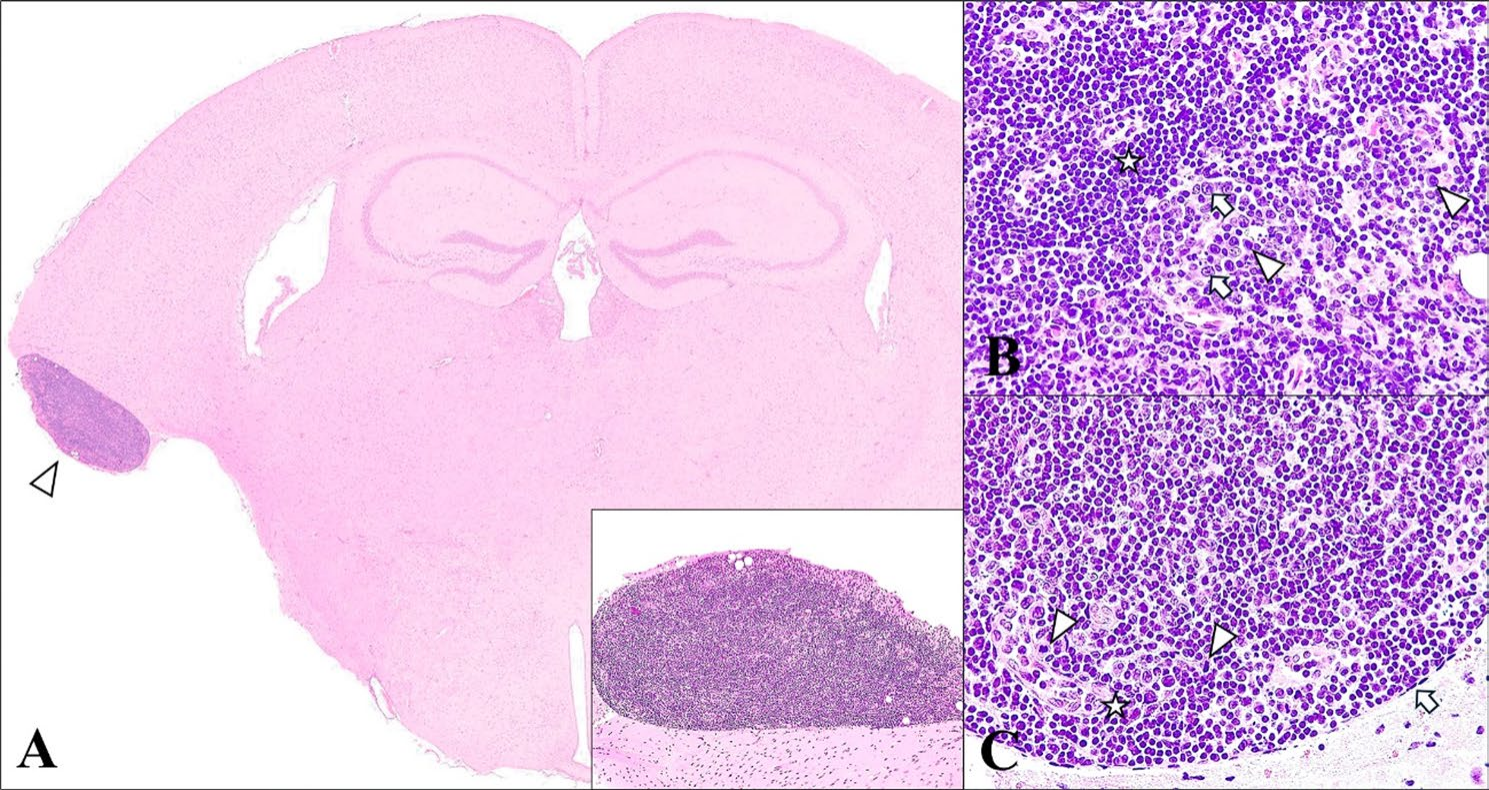
Histopathological description of a round cell tumor in a mouse brain infected with SARS-CoV-2. **A** Leptomeningeal tumor located in the secondary somatosensory cortex (arrowhead) within the diencephalic subdivision. H&E, 1x. Inset: Round cell tumor, ovoid in shape, located in the leptomeninges. H&E, 10x. **B** Moderate presence of mononuclear cells compatible with lymphocytes with indented round nuclei and a variable amount of cytoplasm (star). Large atypical mononuclear cells resembling lymphoblast with round to oval nuclei, larger size, and increased amount of cytoplasm (arrowheads), mixed with pale stained histiocytes (arrows). H&E, 40x. **C** Mild to moderate presence of mitotic figures (arrowheads) and neovascularization (star); the tumor is encapsulated by a thin fibroblastic layer (arrow). H&E, 40x

**Fig. 2:**
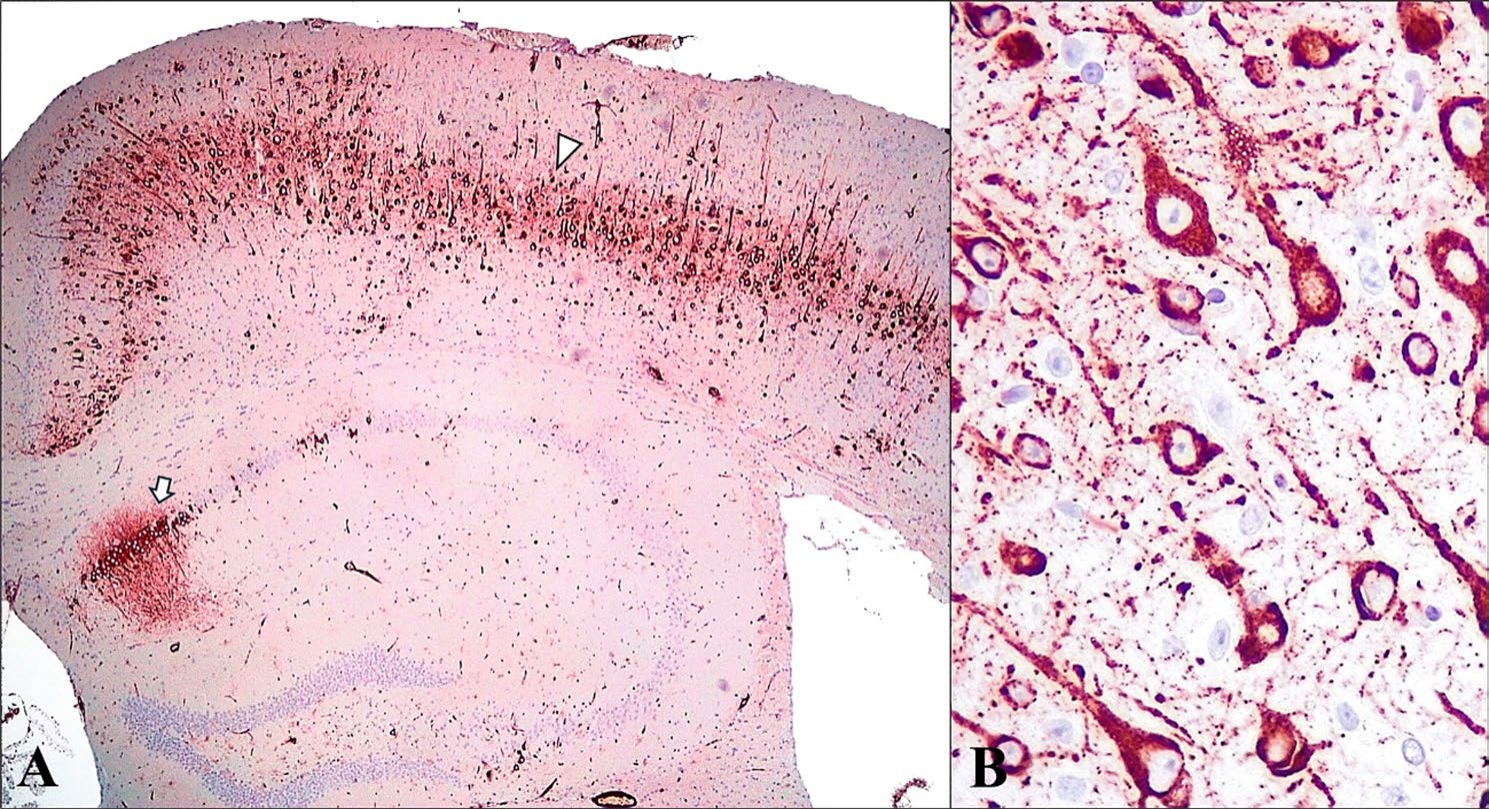
Immunohistochemical detection of viral antigen in a mouse brain infected with SARS-CoV-2. **A** Neuronal SARS-CoV-2 immunoexpression in cortical layers IV-V (arrowhead) and hippocampal pyramidal neurons and stratum radiatum (CA1) (arrow). IHC anti-SARS-CoV-2, 4x. **B** Intracytoplasmic neuronal SARS-CoV-2 immunoexpression. IHC anti-SARS-CoV-2, 40x

Gross evaluation revealed pulmonary congestion with mild dark reddish consolidation areas in the cranial lobes. No other macroscopical alterations were observed in the rest of the organs. Histopathological analysis demonstrated bronchointerstitial pneumonia, with hyperplasia of type II pneumocytes and bronchiolar epithelium, along with mild vascular thrombosis and perivascular oedema. Microscopical examination of the brain revealed the presence of a focal, well-delimitated, and oval shaped, leptomeningeal tumor located in the secondary somatosensory cortex, within the diencephalic subdivision. Its location corresponded to the end of the existing cortical region, suggesting the loss of the lateroventral cortex due to atrophy (Fig. 1A). The tumor measured 1340 μm in length and 590 μm in width and was composed of an abundant number of mononuclear cells resembling lymphoblasts with indented round dense nuclei and a variable amount of cytoplasm. There was a moderate number of large, atypical lymphocytes with round to oval nuclei, larger size, and increased amount of cytoplasm, mixed with abundant histiocytes that exhibited paler staining with prominent nucleoli (Fig. 1B). Additionally, there was a mild to moderate presence of mitotic figures and neovascularization; the tumor was completely encapsulated by a thin fibroblastic layer (Fig. 1C). No evident histopathological findings were observed in the other organs. Furthermore, the presence of tumoral structures in the other organs was discarded, identifying this mass as a primary CNS neoplasm. The brain exhibited diffuse lesions typical of an acute SARS-CoV-2 condition, including meningoencephalitis and neuronal degeneration with cytoplasmic vacuolization and pyknotic, eccentric nuclei. White matter tract myelin sheath vacuolation, increased glial cell proliferation, perivascular lymphocytic cuffs and vasculitis were also observed. Furthermore, the immunohistochemical study of SARS-CoV-2 revealed a widespread distribution of the virus, mainly inside the body and cytoplasmic prolongations (axons and dendrites) of neurons and microglia (Fig. 2).

**Fig. 3:**
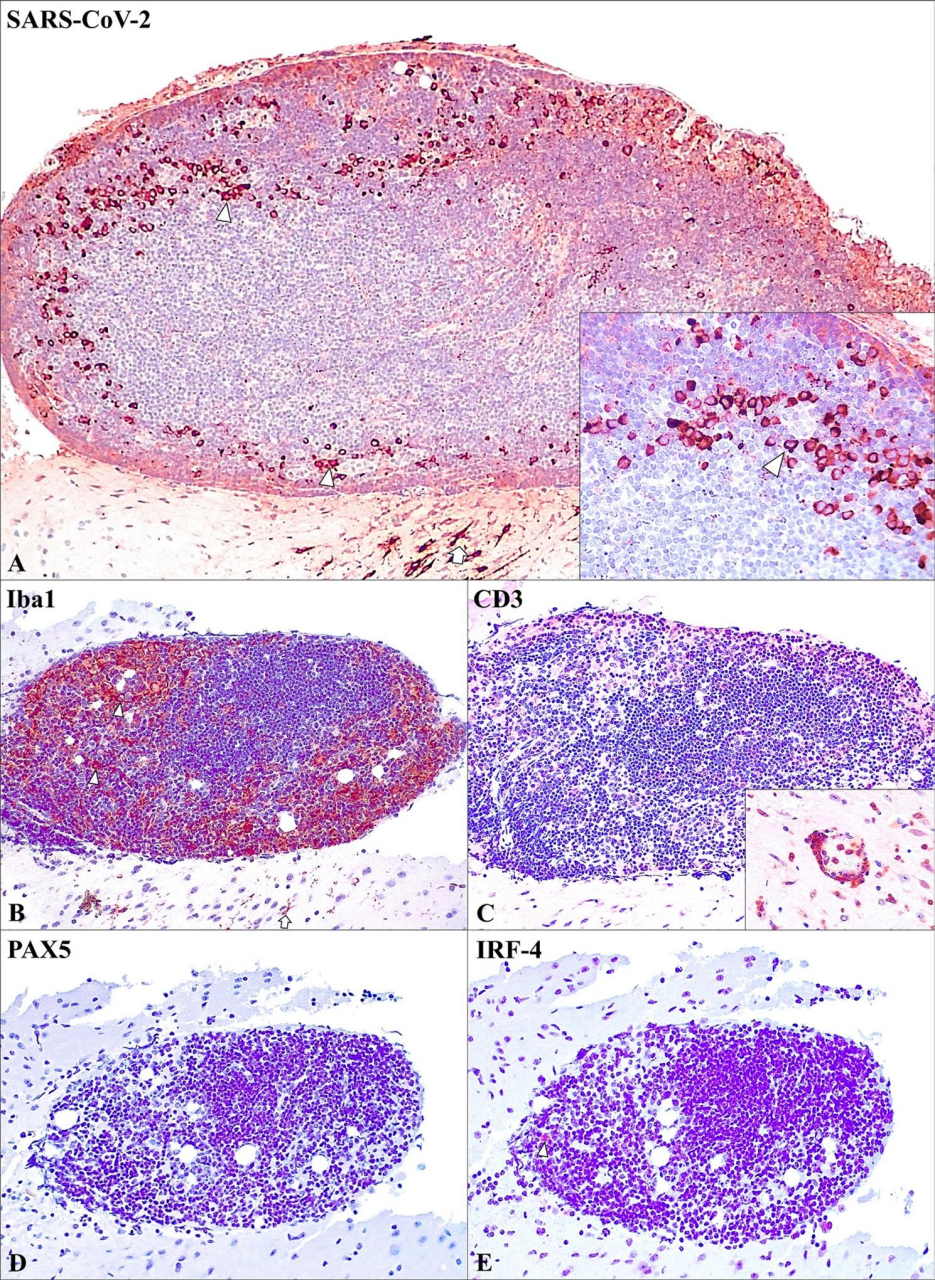
Comparative immunohistochemical analysis (SARS-CoV-2, Iba-1, CD20, PAX5, CD3) in a brain tumor mouse infected with SARS-CoV-2. **A** Moderate immunoexpression in the tumoral histiocytic cells (arrowhead) and adjacent neurons (arrow). IHC anti-SARS-CoV-2, 10x. Inset: SARS-CoV-2 immunoexpression in the histiocytic cells (arrowhead). IHC anti-SARS-CoV-2, 40x. **B** Intense presence of Iba-1 + histiocytic cells, situated peripherically (arrowheads), and neuroparenchymal microglia (arrow); IHC anti-Iba-1, 20x. **C** Absence of CD3 + T cells immunoexpression; IHC anti-CD3, 20x. Inset: CD3 + T cells immunoexpression in the perivascular lymphocytic cuffs; IHC anti-CD3, 40x. **D** Absence of PAX5 + B cells immunoexpression; IHC anti-PAX5, 20x. **E** Almost absence of IRF-4 + cells immunoexpression (arrowhead); IHC anti-IRF-4 +, 20x

Comparative immunohistochemical studies were conducted on the tumor (Fig. 3), indicating the presence of the virus within the cytoplasm of histiocytic-like tumor cells, primarily located at the periphery, adjacent to the infected neurons (Fig. 3A). The immunohistochemical analysis of the tumor revealed a high abundance of Iba-1 + histiocytes (Fig. 3B), primarily localized at the periphery, corresponding to the same cells exhibiting viral immunostaining. The small center of mononuclear cells resembling lymphocytes, were negative to CD3, presenting CD3 + T cells exclusively in the perivascular lymphocytic cuffs within neuroparenchyma (Fig. 3C). These mononuclear cells were also negative to PAX5 immunoexpression (Fig. 3D); a scarce and insignificant number of IRF-4 + cells were found at the periphery (Fig. 3E).

## Discussion and conclusions

Our pathological analysis uncovers an unexpected leptomeningeal neoplasia in k18-hACE2 mouse associated with SARS-CoV-2 infection, reporting for the first time viral tropism in tumor histiocytes. Pathological examination of the tumor revealed a central area composed of mononuclear cells, most likely corresponding to lymphocytes, displaying variable and atypical size; these cells were surrounded by abundant Iba-1 + histiocytic cells. IHC was not able to characterize the lymphocytic constituent of tumor. Although not conclusive, these findings suggest potential differential diagnoses. Histopathological findings may be consistent with histiocyte-rich B-cell lymphoma (HRBCL); however, this diagnosis is unlikely due to the negative PAX5 immunoexpression. IRF-4 negative immunolabeling also discarded multiple myeloma or plasmacytoma formation. Therefore, the most plausible possibility is a lymphoma undergoing transdifferentiation into a histiocytic/dendritic cells neoplasm (HDCN). Related to this, previous studies indicated that PAX5 plays a critical role in maintaining B-cell identity and function. Thus, its reduction or inactivation can lead to the loss of distinctiveness, allowing pro-B-cells to re-acquire the ability to differentiate into macrophages [12], as has been described in the development of histiocytic sarcoma (true histiocytic lymphoma), a type of HDCN [13]. Documented cases of primary brain lymphomas in mice are relatively rare and typically occur in the context of experimental models involving prior genetic or immunological manipulation [7, 14]. To our knowledge, no reports of primary lymphomas in k18-hACE2-mice brain have been documented. However, a limitation of this study was the tumor characterization using an immunohistochemical panel, as the tumor was in its early stage and small-sized, limiting the ability to obtain enough consecutive sections.

The relation between SARS-CoV-2 infection and the development of the incipient tumor is a key point of interest in our study. The qPCR and IHC in brain revealed high viral loads and virus antigen located within the tumoral histiocytes and their adjacent neurons. This fact could be due to the virus entering the cells via type I lectin, specifically CD169, independent of the ACE2 receptor [15]. These findings raise the question of whether the infection may play a specific role in tumor development or contribute to the progression of a pre-existing tumor.

The etiology of lymphoma and the immunological mechanisms remain obscure up to now. Some hypotheses suggest that lymphoma may originate from an imbalance between T helper 1 (Th1) and T helper 2 (Th2) lymphocytes, which favors antibody-dependent immunity [16]. This becomes particularly relevant whether infection occurs after tumor formation, since Th2/Th1 cytokine imbalance has been associated with a higher risk of mortality in COVID-19 [17]. This aligns with studies suggesting that the most common hematological malignancies diagnosed in patients infected with SARS-CoV-2 are lymphomas [6]; in particular, patients with primary DLBCL of the CNS can derive into severe complications after SARS-CoV-2 infection [18]. These data are consistent with the results obtained in experimental studies, which have demonstrated the tropism of SARS-CoV-2 in tumor cell lines derived from hepatoma, glioblastoma [19, 20] and metastatic lung cancer [21] in the brain. These findings suggest that infected tumoral cells may serve as potential viral reservoirs, facilitating viral transport during metastasis and having direct impact on cancer growth and outcome [19–21]. Another study on lung cancer reported that the upregulation of ACE2 may play a key role in tumor progression, as well as increase susceptibility to COVID-19 infection in cancer patients [22]. Given the limitations of our experimental model due to ACE2 receptor overexpression in k18-hACE2 mice strain, the brain appears most susceptible to post-SARS-CoV-2 lesions, showing a strong inflammatory response in animals with high viral loads.

Inversely, it has been shown that SARS-CoV-2 infection can predispose to develop DLBCL [23]. One potential mechanism is the virus-induced upregulation of miR-155, which alters the cell’s activation. The virus-induced ongoing inflammation and cytokines may facilitate B-cell proliferation in these patients [23]. Furthermore, T- [24] and B-cell lymphomas [25] formation have already been reported following COVID19 vaccination, as these malignant lymphomas are occasionally associated with chronic inflammation and continuous stimulation of these T and B-cells [25]. Thus, COVID19 mRNA vaccines may have the capability to overstimulate the immune system and activate an autoimmune response [25]. While instances of lymphomas have been reported following SARS-CoV-2 vaccination, it is plausible that similar or even more pronounced occurrences may arise after SARS-CoV-2 infection.

In addition to the above, recent studies have demonstrated a potential connection between SARS-CoV-2 and cancer, similar to what has been observed with other viruses such as EBV and hepatitis B virus (HBV), which manipulate tumor suppressor protein p53, promoting its degradation [26]. In fact, decreased p53 protein levels were observed, both acutely and in long COVID-19 patients, indicating a potential carcinogenic risk [27]. On the other hand, it appears that the antigenic response to damage-associated molecular pattern (DAMP) and pathogen-associated molecular pattern (PAMP) are similar in infectious disease and cancer. These molecules cause among others a microenvironment of hypoxia that induces the synthesis of lysyl oxidase (LOX) which promotes the invasion and migration of tumor cells [28].

Given the short, seven-day infection period, it is unlikely that the virus directly induces tumor formation, which typically depends on sustained proliferative activity. However, it is worth noting that proliferation rates in mice exceed those in human tumors [29]. Even though factors such as its primary origin, small size, focal and well-defined location, as well as intense viral presence in adjacent neurons and histiocytic neoplastic cells that may suggest a virus-associated inflammatory process, we cannot exclude the possibility that the tumor developed spontaneously or was influenced by other underlying factors.

There is no direct evidence that SARS-CoV-2 causes neoplasm formation. Notwithstanding, immune alterations and inflammation related to COVID-19 could contribute to the environment needed for tumor development in individuals with other risk factors. This remains an area of active research, and further long-term studies are needed to fully understand the potential implications. This work can serve as a starting point to demonstrate the interaction between SARS-CoV-2 infection and neoplasia development.

## Data Availability

Not applicable.

## Abbreviations

CNS: Central nervous system
COVID-19: Coronavirus disease in 2019
DAMP: Damage-associated molecular pattern
Dpi: Day post-infection
DLBCL: Diffuse large B-cell lymphomas
EBV: Epstein-Barr virus
H&E: Hematoxylin-eosin
HBV: Hepatitis B virus
HDCN: Histiocytic/dendritic cells neoplasm
HRBCL: Histiocyte-rich B-cell lymphoma
HIV: Human immunodeficiency virus
IHC: Immunohistochemistry
Th1: Lymphocytes T helper 1
Th2: Lymphocytes T helper 2
LOX: Lysyl oxidase
PAMP: Pathogen-associated molecular pattern
PCNSL: Primary central nervous system lymphoma
SARS-CoV-2: Severe acute respiratory syndrome coronavirus 2
TCID_50_: 50% tissue culture infectious dose
Tg: Transgenic
